# Coxsackievirus B4 Can Infect Human Peripheral Blood-Derived Macrophages

**DOI:** 10.3390/v7112924

**Published:** 2015-11-24

**Authors:** Enagnon Kazali Alidjinou, Famara Sané, Jacques Trauet, Marie-Christine Copin, Didier Hober

**Affiliations:** 1 Laboratoire de virologie EA3610, Faculté de Médecine, Université de Lille, CHU de Lille 59037, France; enagnonkazali.alidjinou@chru-lille.fr (E.K.A.); famara.sane@chru-lille.fr (F.S.); 2 Laboratoire d’immunologie, Faculté de Médecine, Université de Lille, CHU de Lille 59037, France; jacques.trauet@chru-lille.fr; 3 Laboratoire d’anatomie pathologique, Faculté de Médecine, Université de Lille, CHU de Lille 59037, France; marie-christine.copin@chru-lille.fr

**Keywords:** coxsackievirus B, monocyte-derived macrophages, M-CSF, GM-CSF, infection, inflammation, persistence, J0101

## Abstract

Beyond acute infections, group B coxsackieviruses (CVB) are also reported to play a role in the development of chronic diseases, like type 1 diabetes. The viral pathogenesis mainly relies on the interplay between the viruses and innate immune response in genetically-susceptible individuals. We investigated the interaction between CVB4 and macrophages considered as major players in immune response. Monocyte-derived macrophages (MDM) generated with either M-CSF or GM-CSF were inoculated with CVB4, and infection, inflammation, viral replication and persistence were assessed. M-CSF-induced MDM, but not GM-CSF-induced MDM, can be infected by CVB4. In addition, enhancing serum was not needed to infect MDM in contrast with parental monocytes. The expression of viral receptor (CAR) mRNA was similar in both M-CSF and GM-CSF MDM. CVB4 induced high levels of pro-inflammatory cytokines (IL-6 and TNFα) in both MDM populations. CVB4 effectively replicated and persisted in M-CSF MDM, but IFNα was produced in the early phase of infection only. Our results demonstrate that CVB4 can replicate and persist in MDM. Further investigations are required to determine whether the interaction between the virus and MDM plays a role in the pathogenesis of CVB-induced chronic diseases.

## 1. Introduction

Enteroviruses (EV) are small (20–30 nm) non-enveloped positive single-strand RNA viruses that belong to the Picornaviridae family. The genus Enterovirus is very important in medicine and includes several major human pathogens. Group B coxsackieviruses (CVB 1–6) are classified within the Human Enterovirus B (HEV-B) species [1,2]. CVB infections are usually mild and asymptomatic, but they can cause severe acute illnesses [2,3]. In addition, accumulating evidence supports a strong association between these viruses and chronic diseases, such as type 1 diabetes (T1D) [4,5]. The virus is thought to trigger autoimmunity in genetically-predisposed individuals through various mechanisms, including activation of inflammation and persistence, and contributes then to the development of the disease [5]. This pathogenesis strongly relies on the response of the immune system and especially the innate immunity to the virus. Therefore, innate immunity cells are believed to play a major role in the orchestration of this process. The interactions between CVB and innate immunity cells are not well understood.

In T1D patients, EV components have been detected more frequently in peripheral blood mononuclear cells (PBMC) [6,7,8,9], and monocytes were shown to harbor the virus [10].

*In vitro*, monocytes and monocytic cell lines are poorly permissive to CVB. However, these cells can be effectively infected when the virus is previously incubated with non-neutralizing serum or IgGs [11,12]. The antibody-dependent enhancement of CVB4 infection results in the production of high amounts of IFNα [11,12,13,14] and increased levels of other proinflammatory cytokines [15].

Monocytes are produced from progenitors in the blood marrow and usually circulate via the bloodstream to peripheral tissues. In the steady state or in response to inflammation, monocytes migrate into tissues, attracted by various cytokines or necrotic cells, and mature to replenish resident macrophages and dendritic cells (DC) [16]. Macrophages and DC are well known as potent initiators of immune response. These professional antigen-presenting cells are well equipped with several sensors, namely pattern recognition receptors, and can, on the one hand, initiate and control immune responses to invading pathogens and, on the other, maintain tolerance to self-antigens [17].

Human monocyte-derived DCs can be infected by many viruses [18,19,20,21], but were reported to be non-permissive to CVB [22,23].

Macrophages are primarily remarkable phagocytic cells, but they also have a great plasticity that allows them to respond to environmental cues and change their phenotype, depending on the activation state [24]. Macrophages are one of the earliest detectors of danger signals in the host, and their physiology can be highly modified by the mediators of the immune response, especially cytokines. An environmental signal, such as viral infection, can either increase macrophage immune function or give rise to macrophages that are more susceptible to the infection with less antiviral response [24]. Besides being target cells, macrophages could then play a role in the pathogenesis of virus-induced diseases.

In addition to CD8 cytotoxic T lymphocytes, macrophages have been reported to be common in insulitis found in the pancreas of T1D, both at early and later stages of disease progression [25]. Therefore, in the hypothesis of CVB involvement in the pathogenesis of the disease, the role of interactions between macrophages and the virus cannot be ruled out. Such an interaction has not been investigated yet.

*In vitro*, several viruses can infect monocyte-derived macrophages (MDM) [26,27,28] with various outcomes, but no study was reported regarding the infection of these cells with CVB.

In this study, we describe the infection of human MDM by CVB4 and further investigate the inflammation induced by the virus, as well as its persistence in these cells.

## 2. Results

### 2.1. Monocyte-Derived Macrophages

**Figure 1:**
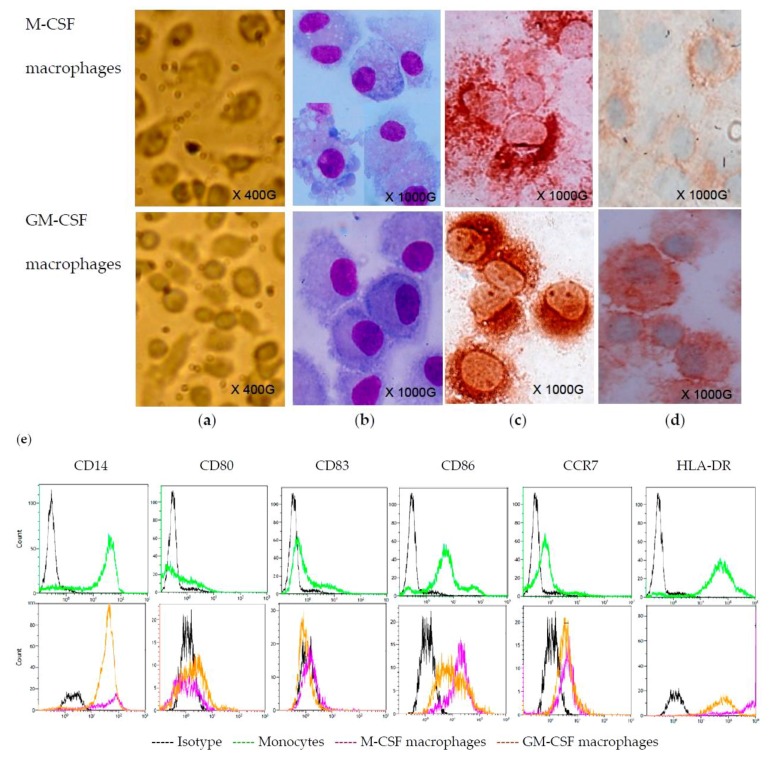
Monocyte-derived macrophages. Monocytes were maintained in serum-free culture medium containing M-CSF or GM-CSF for 7 days. Then cells were directly observed in the culture plate under an inverted microscope (initial magnification X400) (**a**). Cells were cytocentrifuged on slides and stained with MGG (**b**) or labeled with anti-CD68 (**c**) and anti-CD163 (**d**) antibodies by immunocytochemistry (initial magnification X1000). Parental monocytes and derived macrophages were collected and analyzed by flow cytometre (**e**). These observations are representative of three independent experiments.

Monocytes were cultured for seven days in serum-free medium (SFM) containing either M-CSF or GM-CSF. Under these conditions, on Day 7, the cells appeared roughly similar under the microscope, but they were slightly larger in cultures treated with M-CSF (Figure 1a). The staining with MGG displayed that the cells were similar; however, vacuoles or bi-nucleated cells were more common in M-CSF-treated cultures (Figure 1b). M-CSF and GM-CSF-treated cells were macrophages, as they were stained with CD68 antibody (Figure 1c). The macrophages differentiated under these conditions shared the same pattern of staining as regards various markers: CD163+, CD14+, CD80−, CD83−, CD86+, CCR7+ and HLA-DR+ (Figure 1d,e).

### 2.2. CVB4 Can Infect M-CSF-Treated Cells, but not GM-CSF-Treated Cells

**Figure 2:**
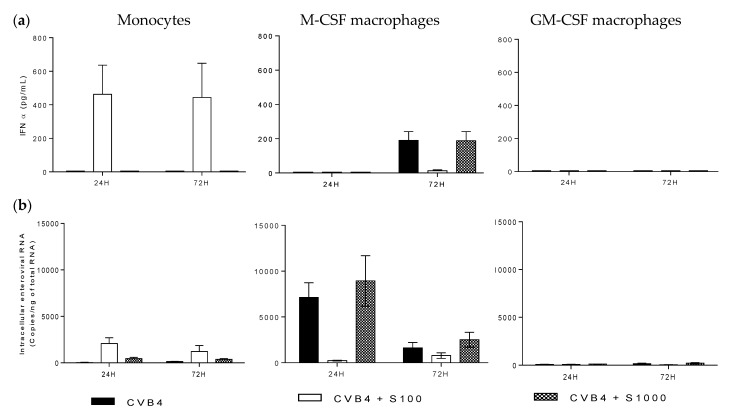
Group B coxsackieviruses 4 (CVB4) can infect M-CSF-induced monocyte-derived macrophages. Monocytes and monocytes treated with M-CSF or GM-CSF for seven days (M-CSF macrophages and GM-CSF macrophages) were inoculated with CVB4 or CVB4 mixed with diluted anti-CVB4 human serum (S100: 1/100 dilution, S1000: 1/1000 dilution). The cell cultures were incubated for 24 h and 72 h, then IFNα levels in the supernatants were measured by using ELISA (**a**), and intracellular viral RNA was quantified by using real-time RT-qPCR (**b**). These results are the mean ± SEM of three independent experiments.

Monocytes and monocyte-derived macrophages were inoculated with CVB4 or CVB4 mixed with non-neutralizing dilutions of human serum. In all of the experiments of this study, the multiplicity of infection (MOI) was one. The cultures were incubated; then, 24 and 72 h post-infection (pi), supernatants were collected for IFNα quantification, and the cells were washed five times with PBS and used for viral RNA quantification. As shown in Figure 2, monocytes did not produce IFNα when they were inoculated with CVB4. However, production of IFNα was obtained when the cells were inoculated with CVB4 mixed with diluted human serum (1/100 dilution); the levels of IFNα reached 462 ± 174 pg/mL and 443 ± 203 pg/mL at 24 h and 72 h pi, respectively, with an interindividual variability (Figure 2a). When M-CSF-treated cells were inoculated with CVB4, no IFNα was detected in the supernatant collected 24 h pi, whereas a significant amount of IFNα was detected 72 h pi (190 ± 51 pg/mL). There was no IFNα in the supernatants of cultures inoculated with CVB4 mixed with human serum (1/100 dilution), but the level was 188 ± 53 pg/mL in supernatants of cultures inoculated with CVB4 mixed with 1000-fold diluted human serum (Figure 2a) and was similar to further dilutions of human serum. Regarding GM-CSF-treated cells, no IFNα was detected in supernatants, whether CVB4 was previously incubated or not with diluted human serum (Figure 2a). IFNα was not detected in supernatants from control cell cultures.

As far as the quantification of intracellular enteroviral RNA was concerned, the levels were very low in monocytes inoculated with CVB4, 85 ± 47 and 146 ± 68 copies/ng of total RNA at 24 and 72 h pi, respectively, and increased significantly when monocytes were inoculated with CVB4 mixed with 100-fold diluted human serum, reaching 2070 ± 626 and 1215 ± 645 copies/ng of total RNA at 24 and 72 h, respectively (see Figure 2b). In M-CSF-treated cells infected with CVB4, the levels of viral RNA (7126 ± 1609 and 1623 ± 601 copies/ng of total RNA at 24 and 72 h) were high. There was no significant increase when the cells were inoculated with CVB4 mixed with 1000-fold diluted human serum (8939 ± 2753 and 2514 ± 801 copies/ng of total RNA) (Figure 2b). In contrast, viral RNA was almost undetectable in GM-CSF-treated cells inoculated with CVB4 or CVB4 mixed with human serum (Figure 2b). No virus was detected in control cell cultures.

Altogether, these results show that CVB4 can infect MDM differentiated in culture medium containing M-CSF and that there is no human serum-dependent enhancement of CVB4 infection of these cells. In contrast, MDM differentiated in culture medium containing GM-CSF were not permissive to CVB4.

**Figure 3:**
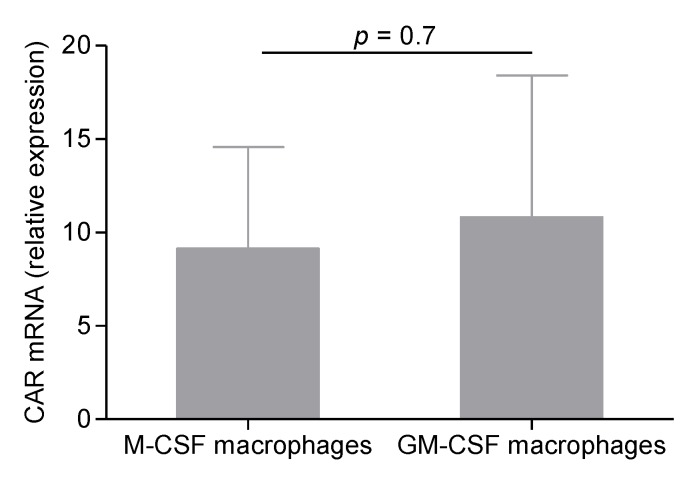
CAR mRNA expression in macrophages. Monocytes and MDM treated with M-CSF or GM-CSF for seven days were collected, and RNA was extracted. CAR mRNA and β-actin were amplified using real-time RT-PCR. CAR relative expression was determined using the 2^−ΔΔ*C*t^ formula, as compared to parental monocytes. These results are the mean ± SEM of three independent experiments.

We hypothesized that GM-CSF-induced MDM were not permissive to CVB4, compared to M-CSF-induced MDM, because of a reduced expression of CAR, the receptor for CVB4. CAR mRNA was then quantified in both cell populations collected after differentiation on Day 7. As shown in Figure 3, the relative expression of CAR was similar in GM-CSF-induced and M-CSF-induced MDM.

### 2.3. CVB4 Can Induce Pro-Inflammatory Cytokines MDM Induced with M-CSF and GM-CSF

**Figure 4:**
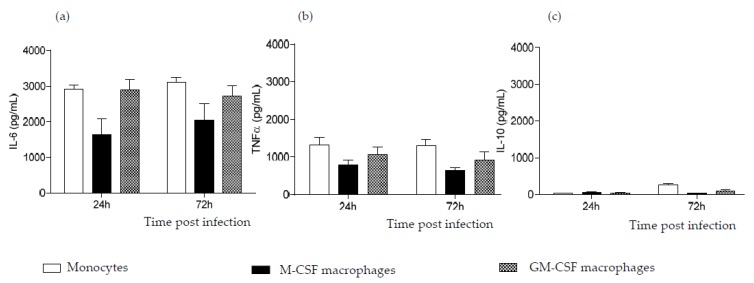
CVB4 can induce pro-inflammatory cytokines in monocyte-derived macrophages (MDM). Monocytes and MDM induced with M-CSF (M-CSF macrophages) or GM-CSF (GM-CSF macrophages) for seven days were inoculated with CVB4. Supernatants were collected at 24 and 72 h post-infection (pi). IL-6 (**a**), TNFα (**b**) and IL-10 (**c**) were measured in supernatants by using ELISA. These results are the mean ± SEM of three independent experiments.

We previously observed that PBMCs were not infected when they were inoculated with CVB4; nevertheless, they can produce high levels of pro-inflammatory cytokines [15]. Therefore, the production of these cytokines in MDM cultures was investigated. Cell cultures were inoculated with CVB4, as described above, and supernatants were collected at 24 and 72 h pi. Like monocytes, both M-CSF- and GM-CSF-induced MDM produced high levels of IL-6, up to 3000 pg/mL in both cell cultures (Figure 4a). CVB4 also induced significant amounts of TNFα in both cell cultures (up to 1200 and 1700 pg/mL in M-CSF- and MG-CSF-induced MDM, respectively) (Figure 4b). On the other hand, CVB4 did not induce any significant production of IL-10 in these cells (Figure 4c). None of these cytokines were detected in control wells.

### 2.4. CVB4 Can Replicate and Persist in M-CSF-Induced MDM

**Figure 5:**
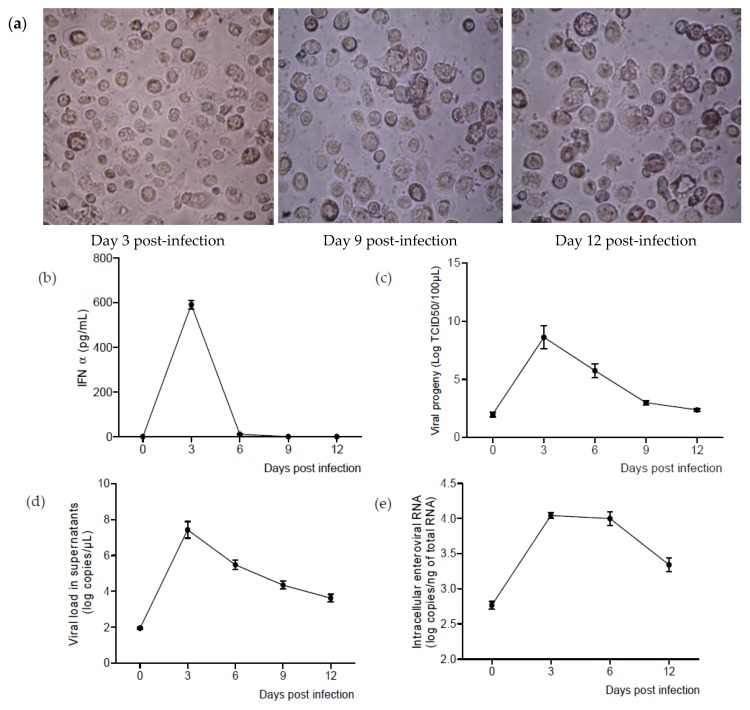
CVB4 can replicate and persist in M-CSF MDM. MDM treated with M-CSF for seven days were inoculated with CVB4 and were cultured for 12 days. The cell viability was assessed using the trypan blue exclusion assay (**a**). In supernatants, the levels of IFNα (**b**), infectious particles (**c**) and enteroviral RNA (**d**) were determined. In cells, enteroviral RNA was quantified by real-time RT-qPCR (**e**). These results are the mean ± SEM of three independent experiments.

In so far as M-CSF-induced MDM were permissive to CVB4 infection, we investigated further the replication and the persistence of the virus in these cells. MDM were inoculated with CVB4, and after 2 h, the cells were extensively washed five times with PBS and then cultured in serum-free medium for 12 days. Supernatants and cells were collected every three days. The viability of cells was assessed during the follow-up (Figure 5a).

High levels of IFNα were detected in supernatants on Day 3 pi. Afterwards, the levels of IFNα decreased dramatically and were almost undetectable on Day 6 pi (Figure 5b).

The levels of infectious viral particles in supernatants were assessed by TCID_50_ determination on HEp-2 cells. The viral titers reached a maximum of 8.6 ± 2 log TCID_50_/100 µL on Day 3 and decreased progressively, but were still around 2.5 log TCD_50_/100 µL on Day 12 (Figure 5c).

The RNA viral load in supernatants was correlated with the viral titers. The maximum value was reached on Day 3 pi (7.4 ± 0.4 log copies/µL) and was 3.6 ± 0.2 log copies/µL on Day 12 (Figure 5d).

Intracellular viral RNA was also detected significantly during the follow-up. The highest levels were around 4 log copies/ng of total RNA and were reached between three and six days pi (Figure 5e).

## 3. Discussion

In this study, we described, for the first time, the infection of human primary macrophages by group B coxsackieviruses. We demonstrated that CVB4 can effectively infect M-CSF-induced MDM, but not GM-CSF-induced MDM. In addition to the production of viral particles, a persistence of viral RNA has been observed in M-CSF-induced MDM cultures.

Previous investigations by our team focused on peripheral blood mononuclear cells (PBMCs) and revealed that among these cells, only monocytes could be infected by CVB *in vitro*, as well as *in vivo* [10]. Monocytes are not spontaneously permissive to the virus; however, the infection can be enhanced by antibodies, as described in previous papers [11,13]. The infection of monocytes with CVB4 can be obtained by pre-incubation of the virus with non-neutralizing dilution of an immune serum before inoculation to cells [11,13]. Enhanced CVB infection of monocytes was shown to rely on both the specific receptor CAR and FCγ receptors, and the target of enhancing antibodies was reported to be the viral protein VP4 [14,29].

However, monocytes are not long-life cells and usually leave the bloodstream after 2–3 days to reach tissues and differentiate into mature cells, such as macrophages [16]. Therefore, the exploration of the interactions between CVB and these cells highly involved in immune response is needed, in the hypothesis of the involvement of the virus in chronic auto-immune diseases, like T1D.

For *in vitro* studies on macrophages, several protocols have been described by researchers to differentiate macrophages from blood monocytes, and usually include the use of: (i) media containing human autologous or heterologous AB serum or fetal bovine serum; or (ii) media containing growth factors, namely M-CSF or GM-CSF [30,31].

In this report, MDM were generated by treating monocytes with a serum-free medium containing either M-CSF or GM-CSF. The phenotypes of cells obtained in both conditions were similar as shown by immunological markers; but surprisingly, only cells treated with M-CSF could be infected by CVB4. It is important to note that the cells were treated for seven days, and then, after washings, they were maintained in culture medium without growth factors. Thus, the opposite patterns of data regarding the infection of MDM in our experiments can be due to some differences between M-CSF-treated cells and GM-CSF-treated cells.

It has been reported that GM-CSF-induced MDM share some transcriptional profiles of classically-activated pro-inflammatory (M1) cells *in vivo*, while those induced with M-CSF tend to replicate alternatively-activated anti-inflammatory (M2) macrophages [30,31,32], but such an association is overestimated. M-CSF and GM-CSF MDM share many features, but are also different regarding the expression of some gene markers and some functions [31,33,34]. A more realistic *in vitro* approach considers MDM generated with M-CSF and GM-CSF as M0 macrophages, and then, M1 originate from M0 induced with GM-CSF or M-CSF in the presence of IFNγ and/or LPS, while M2 macrophages are triggered from M-CSF-induced cells by the presence of cytokines, such as IL-4 or IL-10 [31,35,36]. In so far as the cells were treated with M-CSF or GM-CSF, but not with additional factors, it can be admitted that MDM were M0 macrophages in our experiments.

The discrepancy observed between M-CSF- and GM-CSF-induced MDM regarding CVB4 infection was not due to a reduced transcriptional expression of the specific viral receptor CAR in GM-CSF-treated cells, as shown by quantitative real-time RT-PCR. It remains to be determined whether the receptor is present at the same level at the surface of both M-CSF and GM-CSF cells. Furthermore, in non-polarized cells, it has been reported that a secondary receptor, such as decay-accelerating factor (DAF), is not mandatory for CVB infection, whereas other entry pathways and molecules seem to be critical for entry [37]. Whether these mechanisms of entry are active in M-CSF-induced MDM, but not in GM-CSF-induced MDM cannot be ruled out.

In this study, it has been observed that CVB4 induced the production of high levels of pro-inflammatory cytokines, such as IL-6 and TNα, in both M-CSF MDM and GM-CSF MDM cultures. Since GM-CSF MDM were not infected with CVB4, the production of cytokines was thought to be triggered mainly by surface sensors on these cells, which is in agreement with previous studies in our laboratory. Indeed, it has been shown that inactivated CVB4 can stimulate the production of IL-6, TNFα and IL-12 by monocytes [15], which suggests that a replicative infection of monocytes/macrophages by the virus is not mandatory for inducing inflammation.

In GM-CSF MDM inoculated with CVB4, a low level of viral RNA was detected by sensitive real-time RT-qPCR, but was not associated with the presence of IFNα in supernatants, suggesting that the infection was at a very low level in these cells or was abortive. This hypothesis is in agreement with the demonstration by another team that HIV replication was suppressed in GM-CSF-induced macrophages, but not in M-CSF-induced macrophages [38]. A similar finding was also reported for Mycobacterium tuberculosis, another intracellular pathogen [34].

M-CSF MDM were infected with CVB4, whereas monocytes were infected when the virus was mixed with human non-neutralizing serum. In addition, no enhancement of CVB4 infection (*i.e.*, no increase in FNα or intracellular viral RNA) was obtained in MDM when the virus was mixed with serum, although both monocytes and MDM were reported to express FcγRI and FcγRII [34]. Thus, this suggests that antibodies do not enhance the infection of M-CSF-induced MDM with CVB4.

The investigation of CVB4 persistence in M-CSF-induced MDM displayed that significant levels of infectious particles in supernatants and viral intracellular RNA can be detected up to 12 days (end of the culture), while the cellular response, especially the production of IFNα, was limited to early steps (Day 3). This finding suggests that CVB4 probably overrides the cellular response to establish persistence in MDM. Further studies are needed to understand the mechanisms of interaction between CVB4 and macrophages.

The micro-environment and the phenotype highly impact the physiology of macrophages. Therefore, the relationship between CVB4 and these cells in the pathogenesis of autoimmune diseases, such as T1D, can depend on a specific micro-environment. Indeed, for example, it has been suggested that GM-CSF plays a role in maintaining macrophage populations in a physiological steady state and in controlling the development of autoimmune diseases by regulating the immune response and the immunological tolerance [39].

Whether the phenotype of macrophages recruited in pancreas plays a role in interactions with CVB and in the development of autoimmunity deserves further investigation.

In conclusion, human primary M-CSF-induced MDM, but not GM-CSF-induced MDM, can be infected by CVB4. A productive infection can be obtained, and the virus can persist in these cells. An inflammatory reaction of the cells is observed during the acute phase of the infection. Macrophages can then be considered not only as target cells, but also as reservoirs for CVB4 in tissues, with implications in the pathogenesis of chronic diseases triggered by these viruses.

## 4. Materials and Methods

### 4.1. Virus Stocks

CVB4 E2, the diabetogenic strain of coxsackievirus B4, was used in all experiments. It was kindly provided by Ji-Won Yoon (Julia McFarlane Diabetes Research Center, Calgary, Alberta, Canada) and was propagated in Hep-2 cells (BioWhittaker, Walkersville, MD, USA).

### 4.2. Peripheral Blood Monocyte-Derived Macrophages

Human blood collected from healthy donors was obtained at the Regional Blood Bank (Lille, France). This study was conducted in accordance with the rules of the Declaration of Helsinki of 1975, revised in 2008. Written consent was obtained from donors by the blood bank, and they were informed that the blood would be used for research purposes.

Peripheral blood mononuclear cells (PBMCs) were isolated from buffy coats by density gradient centrifugation using Ficoll-Hypaque^TM^ PLUS (GE Healthcare, Vélizy-Villacoublay, France), as described previously [15]. Cells were resuspended in non-supplemented RPMI 1640 medium, and an average of 10^7^ cells per well (~1.5 million/cm^2^) was seeded in a Falcon® polystyrene six-well plate (Fischer Scientific, Illkirch-Graffenstaden, France). Monocytes from the PBMCs were allowed to adhere to plastic for 2 h at 37 °C, 5% CO2, and non-adherent cells were subsequently removed by aspiration and extensive washing with PBS. Monocytes were cultivated in serum-free medium (SFM) containing either macrophage colony-stimulating factor (M-CSF) or granulocyte-macrophage colony-stimulating factor (GM-CSF) (Peprotech, Neuilly-sur-Seine, France) at 20 ng/mL. The medium was changed on Day 3. On Day 7, the cells were differentiated into macrophages. All media were purchased from Gibco® (Thermo Fischer Scientific, Villebon sur Yvette, France).

### 4.3. Human Serum

Whole blood was centrifugated at 2500 rpm for 15 min, and serum was aliquoted and stored at −20 °C. The level of anti-CVB4 antibodies was determined by the seroneutralization assay, as previously described [15]. Serum with a titer of 256 was used in all experiments.

### 4.4. May–Grunwald–Giemsa Staining and Immunocytochemistry

MDM were detached from the plates by incubation at 4 °C with PBS containing 5 mM EDTA followed by gentle scraping. Then, cells were plated on a slide by cytocentrifugation at 500 rpm for 5 min, and slides were air dried. Cells intended for May-Grunwald-Giemsa (MGG) staining were fixed with ethanol for 5 min and stained using the automated SP-10™ slide maker/stainer (Sysmex, Villepinte, France). For immunocytochemistry purposes, cells were fixed with acetone for 10 min. The FLEX monoclonal mouse anti-human CD68 (clone KP1) antibody was purchased from Dako (Dako, Les Ulis, France), and anti-CD163 monoclonal antibody was supplied by DB Biotech (Kosice, Slovak Republic). The staining was performed using the Ultra View Universal DAB Detection Kit, on the VENTANA® Benchmark XT fully-automated IHC/ISH staining instrument (Roche Diagnostics, Meylan, France).

### 4.5. Flow Cytometry

Monoclonal antibodies directed against human surface markers CD14, CD80, CD83, CD86, CCR7 (Beckman Coulter, Villepinte, France) and HLA-DR (Becton Dickinson Bioscience, Le Pont-de-Claix, France) were used at the supplier’s recommended concentrations. The CD14 antibody was fluorescein isothiocyanate (FITC)-conjugated, whereas CD80, CD83, CD86, CCR7 and HLA-DR antibodies were phycoerythrin (PE)-conjugated. Monocytes and monocyte-derived cells were detached and washed once in PBS. Cell suspensions were incubated at 4 °C for 20 min with the appropriate antibodies. The cells were then fixed in 0.5% paraformaldehyde, washed once with PBS and analyzed by flow cytometry on a Navios Flow cytometer (Beckman Coulter, Inc.).

### 4.6. Quantification of Cytokines

IFNα was measured using the IFNα pan-specific ELISA kit (Mabtech®, Sophia Antipolis, France) that allowed detection of subtypes 1/13, 2, 4, 5, 6, 7, 8, 10, 14, 16 and 17 of IFNα. TNFα, IL-6 and IL-10 were quantified with ELISA kits purchased from Peprotech®. Assays were performed according to the manufacturer’s instructions. The detection ranges were 7–700 pg/mL (IFNα), 23–1500 pg/mL (TNFα), 32–2000 pg/mL (IL-6) and 32–2000 pg/mL (IL-10).

### 4.7. Viral Progeny

The viral titer in supernatants of infected cells was assessed using the end-point dilution assay, and the Spearman–Karber statistical method was used to determine the tissue culture 50% infectious dose (TCID_50_).

### 4.8. RNA Extraction

Cells were previously collected in TriReagent® for cell lysis and stored at −80 °C until RNA extraction. For the extraction of RNA from supernatants, a volume of 250 µL was used. Total RNA was extracted using the TriReagent® RNA isolation reagent/chloroform procedure (Sigma-Aldrich). Extracted RNA was then dissolved in 50 μL of nuclease-free water, quantified with a Nanodrop® spectrophotometer (Thermo Fischer Scientific).

### 4.9. DNase Treatment

A DNA enzymatic digestion was performed on RNA extracts. A volume of 10 μL of RNA was mixed in a tube with 2 μL of DNase RDD10× buffer (Qiagen, Les Ulis, France), 0.2 μL of DNase I (Qiagen), 0.25 μL of RNase inhibitor (Invitrogen/Thermofischer Scientific) and 7.55 μL of nuclease-free water. The tube was incubated at 37 °C for 30 min and at 65 °C for 5 min. The tube was brought to 4 °C and then vortexed.

### 4.10. Enterovirus Real-Time Quantitative PCR

The Affinityscript® QPCR cDNA Synthesis Kit (Agilent, Les Ulis, France) was used for the RNA retro-transcription step on a Perkin Elmer 2400 thermocycler. Quantitative RT-PCR for cDNA amplification was performed with the Brilliant II® QPCR Kit (Agilent) on the Mx3000p® (Stratagene/ Thermofischer Scientific). Oligonucleotides and RT-PCR conditions were previously described [15]. Enterovirus 71 RNA (Vircell, Granada, Spain) was used as the standard for quantification.

### 4.11. Quantification of Coxsackie and Adenovirus Receptor mRNA

CAR mRNA was quantified by real-time RT-qPCR using the SuperScript III Platinum One-Step Quantitative RT-PCR System (Life Technologies/Thermofischer Scientific). The oligonucleotides were already published [40]. The quantification was carried out on the Mx3000p® (Stratagene) with the following program: 50 °C for 15 min, 95 °C for 2 min and 40 cycles of amplification consisting of 15 s at 95 °C and 30 s at 60 °C. The expression of the beta-actin gene was used for normalization. CAR mRNA relative expression in macrophages as compared to parental monocytes was determined with the 2^−ΔΔ*C*t^ formula [41].

### 4.12. Statistical Analysis

Data were presented as the mean ± SEM. Graphs and analyses were performed with GraphPad Prism® V6.0 software. Comparisons were performed with the Mann–Whitney *U* test with the significance set at 0.05.
